# Markov versus quantum dynamic models of belief change during evidence monitoring

**DOI:** 10.1038/s41598-019-54383-9

**Published:** 2019-12-02

**Authors:** Jerome R. Busemeyer, Peter D. Kvam, Timothy J. Pleskac

**Affiliations:** 1 Psychological Brain Sciences, Indiana University, Bloominton, 47405 Indiana USA; 2 Psychology, University of Florida, Gainsville, 32611 Florida USA; 3 Psychology, University of Kansas, Lawrence, 66045 Kansas USA

**Keywords:** Human behaviour, Quantum information

## Abstract

Two different dynamic models for belief change during evidence monitoring were evaluated: Markov and quantum. They were empirically tested with an experiment in which participants monitored evidence for an initial period of time, made a probability rating, then monitored more evidence, before making a second rating. The models were qualitatively tested by manipulating the time intervals in a manner that provided a test for interference effects of the first rating on the second. The Markov model predicted no interference, whereas the quantum model predicted interference. More importantly, a quantitative comparison of the two models was also carried out using a generalization criterion method: the parameters were fit to data from one set of time intervals, and then these same parameters were used to predict data from another set of time intervals. The results indicated that some features of both Markov and quantum models are needed to accurately account for the results.

## Introduction

When monitoring evidence during decision making, a person’s belief about each hypothesis changes and evolves across time. For example, when watching a murder mystery film, the viewer’s beliefs about guilt or innocence of each suspect change as the person monitors the evidence from the ongoing movie. What are the basic dynamics that underlie these changes in belief during evidence accumulation? Here we investigate two fundamentally different ways to understand the dynamics of belief change.

**Figure 1:**
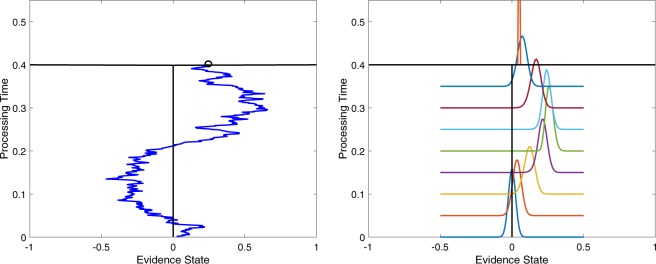
Illustration of Markov (Left) and quantum (Right) processes for evolution of beliefs during evidence monitoring. The horizontal axis represents 101 belief states associated with subjective evidence scale values ranging from 0 to 100 in one unit steps. The vertical axis represents time that has passed during evidence monitoring. The Markov process moves from a belief state located at one value at one moment in time to another belief state at a later moment to produce a sample path across time. At the time of a request for a rating, the current belief state is mapped to a probability rating value (small circle). The quantum process assigns a distribution across the belief states at one moment, which moves to another distribution at a later moment (technically, this figure shows the squared magnitude of the amplitude at each state). At the time of a request for a rating, the current distribution is used to probabilistically select a rating (red vertical line).

The “classical” way of modeling evidence dynamics is to assume that the dynamics follow a Markov process, such as a random walk or a drift diffusion model (see, e.g., references^1–3^). These models are essentially cognitive-neural versions of a Bayesian sequential sampling model in which the belief state at each moment corresponds to the posterior probability of the accumulated evidence^4^. According to one application of this view^5–7^, the decision maker’s belief state at any single moment is located at a specific point on some mental scale of evidence. This belief state changes moment by moment from one location to another on the evidence scale, sketching out a sample path as illustrated in Fig. 1, left panel. At the time point that an experimenter requests a probability rating, the decision maker simply maps the pre-existing mental belief state onto an observed rating response.

A “non-classical” way of modeling evidence dynamics for belief change is to assume that the dynamics follow a quantum process^8–10^. According to one application of this view^11^, the decision maker’s belief state at a moment is not located at any specific point on the mental evidence scale; instead, at any moment, the belief state is indefinite, which is represented by a superposition of beliefs over the mental evidence scale. This superposition state forms a wave that flows across time as illustrated in Fig. 1, right panel (technically, this figure shows the squared magnitude of the amplitude at each state). At the time point that an experimenter requests a judgment, the judge’s indefinite state must be resolved, and the wave is “collapsed” to probabilistically select an observed rating response (red bar at the top of Fig. 1, right panel). Note that we are not proposing that the brain is some type of quantum computer – we are simply using the basic principles of quantum probability theory to predict human behavior. At least one classical neural network model that could implement these principles has been proposed^12^.

**Figure 2:**
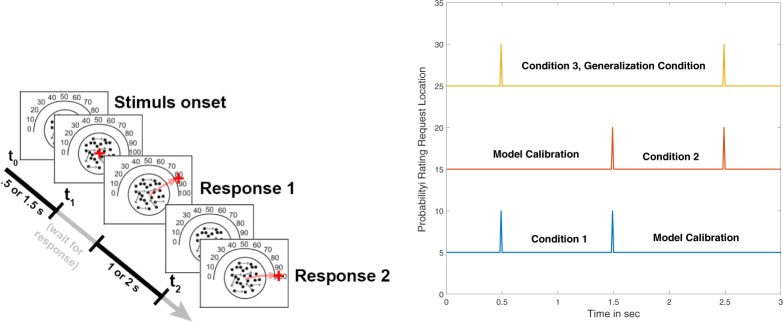
Illustrations of dot motion task with probability rating scale and protocol for experiment (Left), timing of judgments (right). On the left, a judge watches the dot motion and at the appointed time selects a rating response from the semicircle. The judge first watches the dots motion for a period of time, then makes a first choice response, and then observes the dot motion for a period of time again, and then makes a second rating response. On the right, each condition had a different pair of time points for requests for ratings: the time intervals of the first two conditions are contained within condition 3. Conditions 1 and 2 were used to estimate model parameters and then these same model parameters were used to predict the ratings for condition 3.

Previous research^11^ tested and compared the predictions of these two models using a “dot motion” task for studying evidence monitoring^13^. This task has become popular among neuroscientists for studying evolution of confidence (see, e.g., reference^3^). The dot motion task is a perceptual task that requires participants to judge the left/right direction of dot motion in a display consisting of moving dots within a circular aperture (see left panel of Fig. 2). A small percentage of the dots move coherently in one direction (left or right), and the rest move randomly. Difficulty is manipulated between trials by changing the percentage of coherently moving dots. The judge watches the moving dots for a period time at which point the experimenter requests a probability rating for a direction (see Fig. 2, left panel). In the study by Kvam *et al*.^11^, each of 9 participants received over 2500 trials on the dot motion task.

The experimental design used by Kvam *et al*.^11^ included 4 coherence levels (2%, 4%, 8%, or 16%) and two different kinds of judgment conditions. In the choice-confidence condition, participants were given ${t}_{1}=0.5\,{\rm{s}}$ to view the display, and then a tone was presented that signaled the time to make a binary (left/right) decision. After an additional $\Delta t=0.05,0.75,\,or\,1.5\,s$ following the choice, participants were prompted by a second tone to make a probability rating on a 0 (certain left) to 100% (certain right) rating scale (see Fig. 2, left panel). In a confidence-only condition, participants didn’t have to make any decision and instead they simply made a pre-determined response when hearing the tone at time *t*_1_, and then later they made a probability rating at the same total time points *t*_2_ as the choice - confidence condition.

When comparing the choice-confidence condition to the confidence-only condition, a Markov model predicts that the marginal distribution of confidence at time *t*_2_ (pooled across choices at time *t*_1_ for the choice-confidence condition) should be the same between the two conditions. This is a general prediction and not restricted to a particular version of a Markov model. The prediction even holds if the dynamics of the Markov process change between the first and second intervals. We only need to assume that these dynamics are determined by the dot motion information after time *t*_1_ rather than the type of response at time *t*_1_ (decision versus pre-planned response). See pages 247 to 248 in reference^9,11^ for a formal proof. In contrast, a quantum model predicts that these two distributions should be different, and the difference between conditions is called an interference effect.

The predictions for a Markov model are based on the following reasons. Markov processes describe the evolution of the probability distribution over evidence levels across time (see Methods). The probability of a response is computed from the sum of the probabilities of evidence levels associated with that response. Under the confidence - only condition, the probability distribution evolves during the first time interval, and then continues to evolve until the end of the second time interval, which is used to compute the probability of a judgment response. Under the choice-confidence condition, the probability distribution evolves across the first time interval, which is used to compute the probability of a choice. Given the choice that is made at the end of the first time interval, the probability distribution is conditioned (collapsed) on levels consistent with the observed choice. Then this conditional probability distribution continues to evolve until the end of the second interval. The marginal probabilities of the judgments for the choice-confidence condition are computed by summing the joint probabilities of choices followed by judgments over choices. Finally, these marginal probabilities turn out to be equal to the sums across time intervals obtained from the confidence - only condition, because the Markov process obeys the Chapman - Kolmogorov equation, which is a dynamic form of law of total probability^14^.

The contrasting predictions for a quantum model are based on the following reasons. Quantum processes describe the evolution of an amplitude distribution over evidence levels across time (see Methods). The probability of a response is computed from the sum of the squared magnitudes of the amplitudes associated with that response. Under the confidence - only condition, the amplitude distribution evolves during the first time interval, and then continues to evolve until the end of the second time interval, which is used to compute the probability of a judgment response. Under the choice-confidence condition, the amplitude distribution evolves across the first time interval, which is used to compute the probability of a choice. Given the choice that is made at the end of the first time interval, the amplitude distribution is conditioned (collapsed) on levels consistent with the observed choice. Then the conditional amplitude distribution continues to evolve until the end of the second interval. Once again, the marginal probabilities of the judgments for the choice-confidence condition are computed by summing the joint probabilities of choices followed by judgments over choices. However, these marginal probabilities differ from those obtained from the confidence - only condition, because the latter condition produces a sum of amplitudes rather than a sum of probabilities across time intervals, and taking the squared magnitudes of the sum of amplitudes to compute probabilities generates interference terms that violate of the law of total probability.

The results of the experiment strongly favored the quantum model predictions: the interference effect was significant at the group level, and at the individual level, 6 out of the 9 participants produced significant interference effects. Furthermore, parameterized versions of the Markov and quantum models were used to predict both the binary choices and as well as the confidence ratings using the same number of model parameters, and results of a Bayesian model comparison strongly favored the quantum over the Markov model for 7 out of 9 participants.

The current paper presents a new type of test for interference effects. The previous study examined the sequential effects of a binary decision on a later probability judgment; in contrast, the present study examines the sequential effects of a probability rating on a later probability judgment. A binary decision may evoke a stronger commitment, whereas a probability judgment does not force the decision maker to make any clear decision. The question is whether the first probability judgment is sufficient to produce an interference effect like that produced by committing to a binary decision.

Secondly, and more important, the present experiment provided a new generalization test^15^ for *quantitatively* comparing the predictions computed from parameterized versions of the competing models. (The version of the Markov model that we test is a close approximation to a well-established diffusion model^5^). The generalization test provides a different method than the Bayes factor previously used in^11^ for quantitatively comparing the two models because it is based on *a priori* predictions made to new experimental conditions.

A total of 11 participants (8 females, 3 males) were paid depending on their performance for making judgments on approximately 1000 trials across 3 daily sessions (see Methods for more details). Once again, the participants monitored dot motion using 4 coherence levels (2%, 4%, 8%, or 16%) with half of the trials presenting left moving dots and the remaining half of the trials presenting right moving dots. In this new experiment, two probability ratings were made at a pair $({t}_{1},{t}_{2})$ of time points (see Fig. 2, right panel). The experiment included three main conditions: (1) requests for probability ratings at times ${t}_{1}=0.5\,s$ and ${t}_{2}=1.5\,s$, (2) requests for ratings at times ${t}_{1}=1.5\,s$ and ${t}_{2}=2.5\,s$, and (3) requests for ratings at times ${t}_{1}=0.5\,s$ and ${t}_{2}=2.5\,s$. This design provided two new tests of Markov and quantum models.

First of all, we tested for interference effects by comparing the marginal distribution of probability ratings at time ${t}_{2}=1.5\,s$ for condition 1 (pooled across ratings made at time ${t}_{1}=0.5\,s$) with the distribution of ratings at time ${t}_{1}=1.5\,s$ from condition 2. Note that at time ${t}_{1}=1.5\,s$, condition 2 was not preceded by any previous rating, whereas condition 1 was preceded by a rating. Once again, the Markov model predicts no difference between conditions at the matching time points, and in contrast, the quantum model predicts an interference effect of the first rating on the second.

Secondly, this design also provided a strong generalization test for quantitatively comparing the predictions computed from the competing models. The parameters from both models were estimated using maximum likelihood from the probability ratings distributions obtained from the first two conditions for each individual; then these same parameters were used to predict probability rating distribution for each person on the third condition (see Fig. 2). Both models used two parameters to predict the probability rating distributions (see Methods for details): one that we call the “drift” rate that affects the direction and strength of change in the distribution of beliefs, and another that we call the “diffusion” rate that affects the speed of change and dispersion of the distributions. Using maximum likelihood (see Methods for details), we estimated these two parameters from the joint distribution (pair of ratings at 0.5 *s* and 1.5 *s*) obtained from condition 1, and the joint distribution (pair of ratings at 1.5 *s* and 2.5 *s*) from condition 2, separately for each coherence level and each participant. Then we used these same two parameters to predict the joint distribution (pair of ratings 0.5 *s* and 2.5 *s*) obtained from condition 3 for each coherence level and participant.

## Results

The probability ratings were made by moving a cursor (via joystick) across the edge of a semi-circular scale ranging from 0 (certain moving left) to 100 (certain moving right). Ratings for right-moving dots were used directly; but ratings for left-moving dots were rescored as (100 - rating). In this way, a rating of zero represented certainty that dots were moving in the incorrect direction, a rating of 50 represented uncertainty about the direction, and a rating of 100 represented certainty that the dots were moving in the correct direction.

To examine the effect of coherence, we computed the mean and standard deviation of the ratings at *t*_1_ pooled across trials and conditions separately for each participant and coherence level. Averaging these participant means and standard deviations across participants, produced average means (average standard deviations) equal to (53.84(4.07), 59.51(6.33), 66.60(11.08), 79.72(16.81)) for coherence levels 2%, 4%, 8%, 16%, respectively. The prediction of interference derived from the quantum model relies on the assumption that there is second stage processing of information; without second stage processing, the quantum model predicts no interference (cf.^11^). To check this assumption, we tested the effect of the second stimulus interval on the change in probability ratings from time *t*_1_ to *t*_2_ by computing the mean change for each person and coherence level (averaged over conditions). The average mean (average standard deviation) change across participants equaled 1.28(1.87), 1.03(2.94), 2.75(2.42), 2.01(1.86) for coherence levels 2%, 4%, 8%, and 16% respectively. According to a Hotelling T test, this vector of change is significantly different from zero ($F(3,8)=4.5765,p=0.039$), indicating that participants’ judgments moved toward response values in favor of the correct dot motion direction over the second time interval, on average.

**Figure 3:**
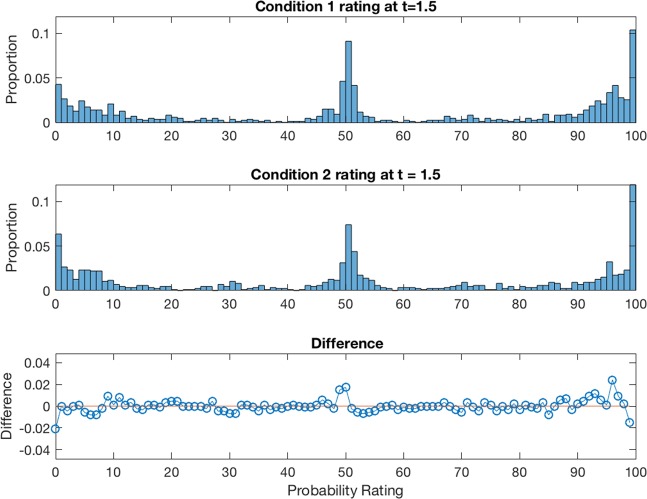
Relative frequency distribution of ratings at 2% coherence level for conditions 1 at ${t}_{2}=1.5$ and condition 2 at ${t}_{1}=1.5$, and interference effect between these conditions. The horizontal axis represents the probability ratings for the correct direction, and the vertical axis represents the proportion assigned to each rating value. Top panel shows results for condition 1, middle panel shows condition 2, and bottom panel shows the difference (top minus middle).

Figure 3 shows the relative frequency distribution of ratings for the lowest (2%) coherence level for conditions 1 at ${t}_{2}=1.5\,s$ and condition 2 at ${t}_{1}=1.5\,s$, and the difference between the two. First note that the ratings tended to cluster into three groups near the end points and middle point of the probability scale. This clustering also occurred with all of the other coherence levels and across participants. Based on the finding that the ratings tended to cluster into three groups, we categorized the data into three levels (L = low ratings from 0 to 33, M = medium ratings from 34 to 66, and H = high ratings from 67 to 100). This also had the benefit of increasing the frequencies within the cells, which was required for the chi - square statistical tests reported next.

### Statistical tests of interval condition effects

First we statistically tested for an interference effect between conditions 1 and 2 using the categorized ratings. For this test, we compared the marginal distribution across the three categories for condition 1 at time ${t}_{2}=1.5\,s$ with the marginal distribution across the three categories for condition 2 at time ${t}_{1}=1.5\,s$. The chi square difference between the marginals for the two conditions was first computed separately for each participant and coherence level, and then summed across participants for each coherence level to produce a total chi square test at each coherence level. The results produced significant differences only for the low coherence levels (the *G*^2^ (chi square statistics) are 38.0, 27.6, 25.4, 28.1, for coherence levels 2%, 4%, 8%, and 16% respectively, and the critical value for $\alpha =0.05$ and $df=11\cdot (3-1)=22$ equals 33.9). Only 3 out of the 11 participants produced significant effects at the low (2%, 4%) coherence levels.

Second, we statistically tested the difference between the joint probability distributions for conditions 1 versus 3 and again for conditions 2 versus 3. These tests were simply manipulation checks. The purpose was to ensure that the generalization test condition was significantly different from each of the calibration conditions. For completeness, we also tested the difference between conditions 1 and 2. In all three cases, there are only two responses, one at *t*_1_ and another at *t*_2_. Only the values of the time points (*t*_2_, *t*_2_) differed across the comparisons. For the test between condition 1 versus 3, we compared the 3 × 3 joint distribution produced by category ratings at time *t*_1_ and *t*_2_ for condition 1 with the 3 × 3 joint distribution produced by category ratings at time *t*_1_ and *t*_3_ for condition 3; likewise for the tests comparing conditions 2 versus 3 and conditions 1 versus 2. The chi square difference between two 3 × 3 joint distributions was first computed for each person separately, and then summed across participants. The results produced significant differences for both the condition 1 versus 3 comparison (*G*^2^ = 116.9, $df=(9-1)\cdot 11=88$, $p=0.0215$) and for the condition 2 versus 3 comparison (*G*^2^ = 192.4, $df=(9-1)\cdot 11=88$, $p < 0.0001$). Five of the 11 participant produced significant differences for these conditions. The difference between conditions 1 versus 2 was also significant (*G*^2^ = 125.8, $df=(9-1)\cdot 11=88$, $p=0.005$).

In summary, the results suggest that interference effects do occur with sequences of judgments, but they are small and occur for only a subset of the participants and coherence conditions. The results also show differences between the calibration conditions (1, 2) and the generalization condition 3, as well as a difference between the two calibration conditions (1, 2). The latter tests simply confirm that our manipulations of time periods were effective.

### Model comparisons

Both models have two parameters: a drift and a diffusion parameter (see Methods). These two parameters were estimated by maximizing the likelihoods of the data from the pair of 3 × 3 joint distributions produced by responses in conditions 1, 2. This was done separately for each participant and coherence level. The discrepancy between data and model predictions was measured using ${G}^{2}=-\,2\cdot LL$, where *LL* symbolizes log likelihood (see Methods), and we computed the difference between models defined as ${G}_{diff}^{2}={G}_{Markov}^{2}-{G}_{quantum}^{2}$. Positive values indicate lower discrepancy (favorable support) for the quantum model. For all four coherence levels, the quantum model produced lower discrepancies for both of the two calibration conditions: the ${G}_{diff}^{2}$, summed across the 11 participants, equaled 675, 703, 595, and 230 for coherence levels 1 through 4 respectively.

A more powerful test of parameterized versions of the Markov versus quantum models was performed for each participant using the generalization criterion method^15^. The parameters estimated from the two calibration conditions were used to compute the predictions for each participant and coherence level for the generalization condition 3. The ${G}_{diff}^{2}$ statistics, summed across participants, were 350, 306, 260, 40 for coherence levels 2%, 4%, 8%, 16% respectively, favoring the quantum model over the Markov model. Eight of the 11 participants produced ${G}_{diff}^{2}$ favoring the quantum model for coherence levels 2%, 4%, and 8%, but only 5 participants produced results favoring the quantum model for coherence level 16%. The results clearly favor the quantum model, but less so for high coherence.

To be complete, we also used parameter estimates from conditions 1 and 3 to predict condition 2; and we used parameter estimates from conditions 2 and 3 to predict condition 1. These results were consistent with the originally planned generalization test using estimates from conditions 1 and 2 to predict to condition 3, and they are reported in the SI.

Tables 1–4 show the predicted and observed frequencies of responses (3 × 3 tables), averaged across participants, for each coherence level. The observed data reveals large frequencies at both Low and High ratings under low coherence conditions. The wave nature of the quantum model provides a way to spread the judgments across both Low and High levels. However, the sample path nature of the Markov model makes it difficult to simultaneously distribute frequencies to both Low and High ratings. To address this problem with the Markov model, a revised Markov model, shown as Markov-V in the tables, is discussed next.

**Table 1:** Observed and predicted distributions for condition 3, averaged across participants, at coherence level 1.

	Obs			Markov			Quantum			Markov-V		
	L2	M2	H2	L2	M2	H2	L2	M2	H2	L2	M2	H2
L1	0.20	0.02	0.07	0.09	0.06	0.03	0.14	0.05	0.04	0.24	0.02	0.01
M1	0.04	0.23	0.05	0.11	0.30	0.16	0.05	0.31	0.07	0.08	0.20	0.09
H1	0.05	0.02	0.32	0.02	0.07	0.15	0.03	0.05	0.25	0.01	0.03	0.32

**Table 2:** Observed and predicted distributions for condition 3, averaged across participants, at coherence level 2.

	Obs			Markov			Quantum			Markov-V		
	L2	M2	H2	L2	M2	H2	L2	M2	H2	L2	M2	H2
L1	0.17	0.02	0.07	0.06	0.05	0.03	0.14	0.05	0.04	0.18	0.03	0.01
M1	0.02	0.22	0.05	0.09	0.28	0.19	0.05	0.29	0.08	0.06	0.19	0.09
H1	0.05	0.02	0.38	0.02	0.07	0.20	0.03	0.05	0.27	0.01	0.03	0.39

**Table 3:** Observed and predicted distributions for condition 3, averaged across participants, at coherence level 3.

	Obs			Markov			Quantum			Markov-V		
	L2	M2	H2	L2	M2	H2	L2	M2	H2	L2	M2	H2
L1	0.12	0.02	0.04	0.04	0.04	0.03	0.10	0.04	0.03	0.12	0.02	0.01
M1	0.02	0.18	0.07	0.06	0.24	0.21	0.04	0.26	0.10	0.05	0.18	0.08
H1	0.03	0.02	0.50	0.01	0.07	0.30	0.02	0.04	0.36	0.01	0.03	0.49

**Table 4:** Observed and predicted distributions for condition 3, averaged across participants, at coherence level 4.

	Obs			Markov			Quantum			Markov-V		
	L2	M2	H2	L2	M2	H2	L2	M2	H2	L2	M2	H2
L1	0.05	0.01	0.03	0.02	0.02	0.02	0.04	0.01	0.01	0.03	0.01	0.01
M1	0.01	0.14	0.05	0.03	0.16	0.14	0.02	0.18	0.04	0.03	0.15	0.07
H1	0.02	0.01	0.68	0.01	0.04	0.59	0.01	0.04	0.66	0.01	0.02	0.67

Note: For example, L1 stands for Low rating after first interval and M2 stands for High rating after second interval. Cells in the upper right off diagonal represent transitions from lower to higher probability ratings during the second interval.

One possible reason for the lower performance of the Markov is that it does not include any variability in the drift across trials. It has been argued that drift rate variability is required to produce accurate fits for the Markov model (see, e.g.^2^). To allow for this variability, we computed the predictions produced by averaging over a normal distribution of drift rates. For this model we estimated three parameters: a mean drift rate, a variance of the drift rate, and a diffusion rate. These three parameters were estimated for each participant and each coherence level (see Methods).

The Markov model with averaging has one more parameter than the quantum model. For this reason, we used the difference between Bayesian information criteria for the two models defined as as $BI{C}_{diff}={G}_{diff}^{2}+p\cdot \,\mathrm{ln}(N)$, where $p=11$ for one extra parameter per participant, and $N=11\cdot 1735$ (number of participants times number of observations per participant). Positive *BIC*_*diff*_ favors the quantum model. The quantum model produced more favorable *BIC*_*diff*_ values at the two lower coherence levels, but the Markov model with averaging was favored at the two higher coherence levels. The *BIC*_*diff*_ values equaled 219, 36, −98, −67 for coherence levels 2%, 4%, 8%, 16% respectively.

For the generalization test condition 3, the Markov model with averaging model produced ${G}_{diff}^{2}$ statistics, summed across participants, equal to 74, 30, −160, −160 for coherence levels 2%, 4%, 8%, 16% respectively (once again, positive values indicate evidence for the quantum model and negative values indicate evidence for the Markov model). The quantum model is favored for the lower coherence levels, but the Markov-V model is favored for the higher coherence levels. For example, 5 participants were favored by the quantum model at the 4% coherence level, but only 2 were favored at the 8% level.

The prediction of the Markov model with drift rate variability are also shown on the right side of Tables 1–4. The predictions of the Markov-V model are much improved over the original Markov model, and the accuracy of the Markov-V model is now comparable to the quantum model.

## Discussion

This article empirically evaluated two different types of dynamic models for belief change during evidence monitoring. According to a Markov process, the decision maker’s belief state acts like a particle that changes from one location to another producing a sample path across time. In contrast, according to the quantum model, the decision maker’s belief state is like a wave spread across the evidence scale that flows across time. These two competing models can be compared using both qualitative tests of properties of each model as well as quantitative comparisons of predictive accuracy.

The Markov and quantum processes make different qualitative predictions regarding interference effects that can occur when a sequence of responses is requested from the decision maker. The Markov process predicts no interference effect, but the quantum process produces interference effects. As mentioned earlier, Kvam *et al*.^11^ examined interference effects under a “choice and then rating” condition. That earlier experiment produced significant interference effects such that confidence was less extreme following a binary decision, and the size of the interference was directly related to the size of the effect of second stage processing, as predicted by the quantum model. The present study examined interference effects under a “ rating and then rating” condition. This new experiment indicated that an interference effect did occur at the low levels of confidence, but the effect was smaller. One way to interpret this difference in empirical results is that using a binary decision for the first measurement may be more effective for “collapsing” the wave function than using a probabilistic judgment for the first measurement, resulting in greater interference between choice and rating responses than for sequential rating responses.

The occurrence of an interference effect is considered as evidence against the basic Markov model. One could make ad hoc assumptions about how the choice at time *t*_1_ changes the dynamics after that response. For example, one possibility is that a decision at time *t*_1_ produces a bolstering effect that increases the confidence in favor of the decision. However, the interference effect found by Kvam *et al*.^11^ went in the opposite direction, contrary to this ad hoc explanation for their experiment. Moreover, Kvam *et al*.^11^ examined a wide range of ad hoc assumptions which failed to account for their results.

The present experiment is unique in the way the two models were quantitatively compared. A generalization criterion method was used, which allowed us to examine not just how well the models fit the data, but rather how well they could predict data obtained from a new and different condition. Using this method, the parameters of the models were estimated from conditions 1 and 2 and these same parameter estimates were then used to predict a new condition 3. The results of the present experiment indicated that the quantum model produced more accurate predictions for low levels of confidence, but the Markov model (with drift rate variability) produced more accurate predictions for high levels of confidence. Together these results suggest that neither process alone, quantum or Markov, is sufficient to account for all conditions and all participants.

Rather than treating Markov and quantum models as mutually exclusive, an alternative idea is that a more general hybrid approach is needed, one that integrates both quantum and Markov processes. The quantum model used in the present work is viewed as a “closed system” quantum process with no external environmental forces. Technically, only Schrödinger evolution is used (see Methods). However, it is possible to construct an “ open system” quantum process where a person’s belief state partially decoheres as a result of interaction with a noisy mental environment. Technically, Lindblad terms are added to the evolution equation, which forms a more general master equation, producing a combined quantum-Markov process^10,16,17^. Open system quantum models start out in a coherent quantum regime (represented by a density matrix with off diagonal terms, see Methods) and later decoheres into a classical Markov regime (represented by density matrix with no off diagonal terms, see Methods)^16^. In fact, previous work^18^ compared Markov and quantum models with respect to their predictions for both choice and decision time: When a closed system quantum model was compared to the Markov model, there was a slight advantage for the Markov model; however, when an open system quantum model was used, the quantum model produced a small advantage. For our application, we would need to speculate that the speed of decoherence (i.e., change from a quantum to Markov regime) depends on the experimental coherence level, but the development of a specific open system quantum model for belief change is left for future research.

## Methods

### Participants

A total of 11 Michigan State University (8 female, 3 male) students were recruited for the study – 1 additional participant began the study but was dropped for failing to complete all sessions of the study. Each of the 11 remaining participants completed 3 sessions of the study and were paid $10 per session plus an additional bonus based on the accuracy of their confidence ratings – up to $5 based on how close they were to the “100% confident in the correct direction” responses on each trial. Each participant competed approximately 1000 trials of the task across all sessions. The Michigan State University institutional review board approved the experiments; all experiments were performed in accordance with relevant named guidelines and regulations; informed consent was obtained from all participants.

### Task

In the task, participants viewed a random dot motion stimulus where a set of dots were presented on screen. Most of these dots moved in random directions, but a subset of these dots was moving coherently to either the left or the right side of the screen. The dots were white dots on a black background which composed a circular aperture of approximately 10 visual degrees in diameter. The display was refreshed at 60 Hz and dots were grouped into 3 dot groups that were presented in sequence (group 1, 2, 3, 1, 2, 3,) and displaced by a quarter of a degree every time they appeared on screen, for apparent motion at 5 degrees per second. When prompted, participants indicated their confidence that the dots were moving left or right on a scale from 0 (certain that they are moving left) to 100 (certain that they are moving right). They entered their responses by using a joystick to move the cursor across the edge of a semicircular confidence scale like the one shown in Fig. 2.

To begin each trial, participants pressed the trigger button on a joystick in front of them while the cursor – presented as a crosshair – was in the middle of the screen. The random dot stimulus then appeared on the screen, with 2%, 4%, 8%, or 16% of the dots moving coherently toward one (left vs. right) direction. After 500 ms or 1500 ms, participants were prompted for their first probability judgment response with a 400 Hz auditory beep. They responded by moving the cursor across the semicircular confidence scale at the desired probability response. Since participants were using a joystick, the cursor naturally returned to the center of the screen after this initial response. Once the first response had been made, the stimulus remained for an additional 1000 or 2000 ms before a second auditory beep prompting the second probability response. Participants made their second response in the same way as the first. This resulted in a possible on-screen stimulus time of 1500 or 2500 ms plus the time it took to respond.

The stimulus coherence was halted while participants entered their response, but the stimulus was still on-screen - so there were dots just randomly appearing and disappearing but there was no useful information until they entered their rating (which participants were told, so that they wouldn’t try to sample more information between the beep and their response).

After each trial, participants received feedback on what the correct dot motion direction was and how many points they received for their confidence responses on that trial. We recorded the amount of time it took participants to respond after each auditory beep, the confidence responses they entered, the number of points received for the trial, and the stimulus information (coherence, direction, beep times). Everything was presented and recorded in Matlab using Psychtoolbox and a joystick mouse emulator^19^.

### Procedure

Participants volunteered for the experiment by signing up through the laboratory on-line experiment recruitment system, which included mainly Michigan State students and the East Lansing community. Upon entering the lab, they completed informed consent and were briefed on the intent and procedures of the study. The first experimental session included extensive training on using the scale and joystick, including approximately 60 practice trials on making accurate responses to specific numbers, single responses to the stimulus, making two accurate responses to numbers in a row, and making two responses to the stimulus (as in the full trials).

Subsequent experimental sessions started with 30–40 “warm-up’ ‘ trials that were not recorded. After training or warm-up, participants completed 22 (first session) or 28 (subsequent sessions) blocks of 12 trials, evenly split between confidence timings and stimulus coherence levels. The timing and coherence manipulations were random within-block, so each block of 12 trials included every combination of coherence (4 levels) and confidence timing. After every block of trials, they completed 3 test trials where they were asked to hit a particular number on the confidence scale rather than respond based on the stimulus. This was included to get a handle on how accurate and precise the participants could be when using the joystick and understand how much motor error was likely factoring into their responses. Ultimately, motor error was controlled by grouping responses into the three main confidence levels - motor error was far less than the distance between confidence categories on the physical scale.

At the conclusion of the experiment, participants were debriefed on its intent and paid $10 plus up to $5 according to their performance. Performance was assessed using a strictly proper scoring rule^20^ so that the optimal response was to give a confidence response that reflected their expected accuracy. Participants received updates on the number of points they received at the end of each block of the experiment, including at the end of the study.

### Mathematical Models

There are different types of Markov processes that have been used for evidence accumulation. One type is a discrete state and time Markov chain^21^, another type is a discrete state and continuous time random walk process^22^, another type is a continuous state and discrete time random walk^1,23^, and fourth type is a continuous state and continuous time diffusion process^24^. However, the discrete state models converge to make the same predictions as the diffusion process when there are a large number of states and the step size approaches zero^25^.

Like the Markov models, there are different types of quantum processes. One type is a discrete state continuous time version^10,11^ and another type is a continuous state and time version^8^.

To facilitate the model comparison, we tried to make parallel assumptions for the two models. The use of a discrete state and continuous time version for both the Markov and quantum models serves this purpose very well. Additionally, a large number of states were used to closely approximate the predictions of continuous state and time processes.

For both models, we used an approximately continuous set of mental belief states. The set consisted of $N=99$ states $j\in \{1,\ldots ,99\}$, where 1 corresponds to a belief that the dots are certainly not moving to the right (i.e., a belief that they are certainly moving to the left), 50 corresponds to completely uncertain belief state, and 99 corresponds to a belief that the dots are certainly moving to the right. We used 1–99 states instead of 0–100 states because we categorized the states into three categories and 99 can be equally divided into three sets. For a Markov model, the use of $N=99$ belief states produces a very closely approximation to a diffusion process.

For the Markov model, we define ${\phi }_{j}(t)$ as the probability that an individual is located at a belief state *j* at time *t* for a single trial, which is a positive real number between 0 and 1, and $\sum \,{\phi }_{j}(t)=1$. These 99 state probabilities form a *N* × 1 column matrix denoted as $\phi (t)$. For the quantum model, we define ${\psi }_{j}$ as the amplitude that an individual assigns to the belief state located a evidence level *j* on a single trial (the probability of selecting that belief state equals $|{\psi }_{j}{|}^{2}$). The amplitudes are complex numbers with modulus less than or equal to one, and $\sum \,|\psi {|}^{2}=1$. The 99 amplitudes form a *N* × 1 column matrix denoted as $\psi (t)$. Both models assumed a narrow, approximately normally distributed (mean zero, standard deviation = 5 steps in the 99 states), initial probability distribution at the start (*t* = 0) of each trial of the task.

The probability distribution for the Markov process evolves from $\tau$ to time $\tau +t$ according to the Kolmogorov transition law $\phi (t+\tau )=T(t)\cdot \phi (\tau )$, where $T(t)$ is a transition matrix defined by the matrix exponential function $T(t)=exp(t\cdot K)$. Transition matrix element *T*_*ij*_ is the probability to transit from the state in column *j* to the state in row *i*. The intensity matrix *K* is a *N* × *N* matrix defined by matrix elements ${K}_{ij}=\alpha  > 0$ for $i=j-1$, ${K}_{ij}=\beta  > 0$ for $i=j+1$, ${K}_{ii}=-\,\alpha -\beta$, and zero otherwise. The amplitude distribution for the quantum process evolves from $\tau$ to time $\tau +t$ according to the Schrödinger unitary law $\psi (t+\tau )=U(t)\cdot \psi (\tau )$, where $U(t)$ is a unitary matrix defined by the matrix exponential function $U(t)=exp(\,-\,i\cdot t\cdot H)$. Unitary matrix element *U*_*ij*_ is the amplitude to transit from the state in column *j* to the state in row *i*. The Hamiltonian matrix *H* is a *N* × *N* Hermitian matrix defined by matrix elements ${H}_{ij}=\sigma$ for $i=j+1$, ${H}_{ij}={\sigma }^{\ast }$ for $i=j-1$, ${H}_{ii}=\mu \cdot \frac{i}{N}$, and zero otherwise.

For both models, we mapped the 99 belief states to 3 categories using the following three orthogonal projection matrices *M*_*L*_, *M*_*M*_, and *M*_*H*_. Define **1** as a vector of 33 ones, and define **0** as a vector of 33 zeros. Then ${M}_{L}=diag[{\bf{1}},{\bf{0}},{\bf{0}}]$, ${M}_{M}=diag[{\bf{0}},{\bf{1}},{\bf{0}}]$ ${M}_{H}=diag[{\bf{0}},{\bf{0}},{\bf{1}}]$. Finally, define $\parallel X{\parallel }^{1}$ as the sum of all the elements in the vector *X*, and $\parallel X{\parallel }^{2}$ as the sum of the squared magnitude of the elements in the vector *X*.

For the Markov model, the probabililty of reporting rating *l* at time *t*_2_ equals

1 $$p(R({t}_{2})=l)=\parallel {M}_{l}\cdot T({t}_{2}-{t}_{1})\cdot T({t}_{1})\cdot \phi (0){\parallel }^{1}.$$

and for the quantum model, the probabililty of reporting rating *l* at time *t*_2_ equals

2 $$p(R({t}_{2})=l)=\parallel {M}_{l}\cdot U({t}_{2}-{t}_{1})\cdot U({t}_{1})\cdot \psi (0){\parallel }^{2}.$$

For the Markov model, the joint probability of choosing category *k* at time *t*_1_ and then choosing category *l* at time *t*_2_ equals

3 $$p(R({t}_{1})=k,R({t}_{2})=l)=\parallel {M}_{l}\cdot T({t}_{2}-{t}_{1})\cdot {M}_{k}\cdot T({t}_{1})\cdot \phi (0){\parallel }^{1}.$$

For the quantum model, the joint probability of choosing category *k* at time *t*_1_ and then choosing category *l* at time *t*_2_ equals

4 $$p(R({t}_{1})=k,R({t}_{2})=l)=\parallel {M}_{l}\cdot U({t}_{2}-{t}_{1})\cdot {M}_{k}\cdot U({t}_{1})\cdot \psi (0){\parallel }^{2}.$$

As can be seen in Eqs. 3 and 4, both models include a “collapse” on the choice at time *t*_1_. But this turns out to have no effect for the Markov model. To test interference, we sum Eqs. 3 and 4 across *k* and compare these sums with Eqs. 1 and 2 respectively.

The Markov model required fitting two parameters: a “drift” rate parameter $\mu =\frac{\alpha }{\alpha +\beta }$ and a diffusion rate parameter $\gamma =(\alpha +\beta )$. The quantum model required fitting two parameters: a “drift” rate parameter *μ*, and a “diffusion” parameter *σ*. The parameter *μ* must be real, but *σ* can be complex. However, to reduce the number of parameters, we forced *σ* to be real. The model fitting procedure for both the Markov and the quantum models entailed estimating the two parameters from conditions 1 and 2 separately for each participant and each coherence level using maximum likelihood.

The Markov-V model used an approximately normal distribution of “drift” rate parameters. This model required estimating three parameters: *μ* representing the mean of the distribution of drift rates, *σ* representing the standard deviation of the drift rates, and $\upsilon$ representing the diffusion rate. These were also estimated using maximum likelihood. The predictions for the Markov-V model were then obtained from the expectation

5 $$p(R({t}_{1})=k,R({t}_{2})=l)=\sum _{\mu }\,p(\mu )\cdot p[R({t}_{1})=k,R({t}_{2})=l|\mu ]$$

where *p*(*μ*) is a discrete approximation to the normal distribution.

A master equation can be formed by combining Schrödinger and Lindblad evolution operators. The master equation operates on a state defined by a density matrix, $\rho (t)$, which is formed by the outer product $\rho (t)=\psi (t)\cdot \psi {(t)}^{\dagger }$. A coherent quantum state has a density matrix containing off diagonal terms; a classical state has a density matrix with only diagonal terms. The system guided by the master equation initially starts out in a coherent quantum state, but then decoheres toward a classical state.

## Supplementary information

SI

## Data Availability

The datasets and computer programs used in the current study are available at http://mypage.iu.edu/jbusemey/quantum/DynModel/DynModel.htm for models and https://osf.io/462jf/ for data.
