# Supplementary material for: Markov versus quantum dynamic models of belief change during evidence monitoring
Source: Sci Rep. 2019 Dec 2;9:18025. doi: 10.1038/s41598-019-54383-9 (PMC6889126; doi:10.1038/s41598-019-54383-9)
Supplement: Supplementary file 1 — SI [file 41598_2019_54383_MOESM1_ESM.pdf]

Markov versus quantum dynamic models of belief change during evidence monitoring

Jerome R. Busemeyer Peter D. Kvam and Timothy J. Pleskac

Model comparisons using generalization test

G-square lack of fit measures
Generalization from Conditions 1,2 to Condition 3
Q = quantum M = Markov MA = Markov Average

	Coh 1<br>Q Cond 12	Coh 1<br>Q Cond 3	Coh 1<br>M Cond 12	Coh 1<br>M Cond 3
subj 1	74.1312	35.5759	186.9067	98.2573
2	35.0784	14.7249	74.5761	30.9777
3	56.636	7.2796	131.6318	47.3554
4	30.7575	2.1042	27.3051	1.6754
5	20.8149	28.3828	67.7595	57.2188
6	31.9456	19.3412	118.9699	83.6076
7	136.4175	74.3255	263.8386	127.2307
8	109.6085	53.4395	236.4428	138.9655
9	61.6647	49.602	149.8315	104.9442
10	89.5102	53.3343	116.9164	56.214
11	52.2557	21.0636	28.0168	10.7373
avg	63.5291	32.6521	127.4723	68.8349

	Coh 2<br>Q Cond 12	Coh 2<br>Q Cond 3	Coh 2<br>M Cond 12	Coh 2<br>M Cond 3
sub 1	76.9565	24.7135	206.8257	89.6812
2	33.6098	21.6565	45.0722	37.1052
3	55.252	20.5206	110.2128	50.5024
4	31.6222	18.0429	17.6012	8.671
5	38.4275	24.9007	88.0218	31.4422
6	51.4636	22.4943	126.0658	72.246
7	121.738	88.4715	228.9264	137.1209
8	113.9083	38.3707	263.6973	118.8082
9	54.7353	41.097	160.4664	122.3198
10	81.7244	88.6848	135.6634	98.4648
11	55.8125	34.3844	35.9087	25.017
avg	65.0227	38.4852	128.9511	71.9435

	Coh 3<br>Q Cond 12	Coh 3<br>Q Cond 3	Coh 3<br>M Cond 12	Coh 3<br>M Cond 3
sub 1	94.1314	48.2678	203.4847	109.2475
2	38.9827	59.3446	33.5332	20.9041
3	44.4089	6.271	89.6092	32.504
4	2.2358	9.0944	21.5303	6.9794
5	47.5429	31.2694	63.3235	31.7737
6	32.0057	13.8374	110.6935	73.87
7	149.7536	98.7115	258.6728	138.09
8	93.8928	42.379	202.8778	111.094
9	89.0338	44.0839	189.5792	92.1763

	10	88.5877	53.0477		132.4152	57.3788
	11	72.8716	54.5232		43.028	38.8206
avg		68.4952	41.8936		122.613	64.8035

		Coh 4	Coh 4		Coh 4	Coh 4
		Q Cond 12	Q Cond 3		M Cond 12	M Cond 3
sub 1		72.8393	51.218		125.6868	61.5993
	2	0	13.0306		0	13.0306
	3	68.4595	63.59		100.0405	29.5324
	4	23.0069	21.3566		25.0537	16.8915
	5	19.197	18.5989		4.8917	14.6287
	6	37.2783	9.2176		147.8849	72.4532
	7	42.8513	58.1307		40.643	50.9093
	8	105.8491	70.1509		148.5336	87.7369
	9	92.8869	89.4048		70.4763	67.304
	10	122.8929	66.824		136.5376	77.9292
	11	52.0058	26.2359		67.261	38.757
avg		57.9334	44.3416		78.819	48.252

G-square lack of fit measures
Generalization from Conditions 2,3 to Condition 1
Q = quantum M = Markov MA = Markov Average

	Coh 1<br>Q Cond 12	Coh 1<br>Q Cond 3		Coh 1<br>M Cond 12	Coh 1<br>M Cond 3
subj 1	91.305	33.6271		192.8874	84.2498
2	47.905	19.3669		84.5446	37.4529
3	32.5602	44.1738		116.996	85.8656
4	14.4068	27.0011		23.9716	17.278
5	35.2661	14.166		77.7829	43.0633
6	53.986	8.7238		150.9413	50.6038
7	105.66	93.0464		239.4512	149.4331
8	111.5502	59.0763		230.6375	140.9429
9	68.5606	30.5684		171.0242	79.2871
10	107.5682	67.016		109.2946	67.0212
11	39.2146	26.9149		32.4665	18.7143
avg	64.3621	38.5164		129.9998	70.3556

	Coh 2<br>Q Cond 12	Coh 2<br>Q Cond 3		Coh 2<br>M Cond 12	Coh 2<br>M Cond 3
sub 1	70.8954	44.8358		175.1068	117.8851
2	53.2143	20.4424		76.5161	22.8936
3	29.9242	29.9634		109.3202	71.7062
4	2.9572	45.5883		20.8756	15.5736
5	33.8927	18.8257		85.8078	36.4697
6	23.1685	13.0542		125.7251	65.2941
7	119.1684	80.3705		233.631	127.5168
8	91.7669	71.9554		217.0617	166.5339
9	99.2605	22.1346		195.3561	76.0823
10	96.5441	63.1582		165.1877	67.6169
11	62.4156	57.8577		41.494	16.5853
avg	62.1098	42.5624		131.462	71.287

	Coh 3<br>Q Cond 12	Coh 3<br>Q Cond 3		Coh 3<br>M Cond 12	Coh 3<br>M Cond 3
sub 1	85.1149	61.176		196.3663	124.0161
2	101.3888	17.9656		77.9769	32.5173
3	47.1136	33.4733		94.592	46.8349
4	9.0976	4.5728		16.4171	12.8129
5	39.0867	38.9475		44.6045	47.0467
6	35.9183	22.6089		126.1088	56.8382
7	152.9026	81.782		235.4621	140.2219
8	92.2281	45.3538		209.0051	106.0149
9	98.1316	45.1404		185.9869	94.2958

	10	72.9884	53.1504		133.5982	50.508
	11	66.6737	48.9728		46.8985	22.6147
avg		72.7858	41.1949		124.2742	66.7019

		Coh 4 Q Cond 12	Coh 4 Q Cond 3		Coh 4 M Cond 12	Coh 4 M Cond 3
sub 1		57.8891	49.7332		103.6559	93.5173
	2	13.0306	0		13.0306	0
	3	99.2413	44.1162		101.2508	56.0464
	4	21.4679	20.5309		12.8727	17.6174
	5	15.1147	22.3747		10.2168	8.1575
	6	29.7721	29.0698		134.3918	88.9605
	7	56.1278	31.4943		76.4425	23.0187
	8	93.7464	61.3638		155.5474	77.2018
	9	145.3498	40.2623		113.6326	34.6034
	10	113.4922	81.9398		138.7544	83.6611
	11	42.6087	26.55		93.5278	35.8057
avg		62.531	37.0395		86.6657	47.1445

G-square lack of fit measures
Generalization from Conditions 1,3 to Condition 2
Q = quantum M = Markov MA = Markov Average

	Coh 1	Coh 1		Coh 1	Coh 1
	Q Cond 12	Q Cond 3		M Cond 12	M Cond 3
subj 1	66.7324	58.7316		145.7168	131.0914
2	30.6326	34.5138		56.4767	68.8858
3	43.791	27.5852		115.3882	90.2772
4	4.8018	28.0161		12.0678	23.7122
5	41.4715	7.981		76.9789	44.1681
6	27.2682	20.6691		118.1017	83.6792
7	130.6887	68.3082		253.2776	140.0277
8	120.2728	50.7578		245.4689	125.2128
9	80.8429	50.2925		145.6679	103.5123
10	47.162	23.7357		113.7155	61.7602
11	44.1214	22.129		35.2355	17.1013
avg	57.9805	35.7018		119.8269	80.8571

	Coh 2	Coh 2		Coh 2	Coh 2
	Q Cond 12	Q Cond 3		M Cond 12	M Cond 3
sub 1	67.539	48.0601		173.8111	120.2381
2	50.354	24.1062		54.8559	46.0888
3	32.5469	26.8264		101.4996	82.5035
4	18.6016	30.9729		16.2995	16.0638
5	33.0035	20.4803		54.9959	65.8468
6	21.4423	15.0782		121.7507	68.3912
7	124.09	75.8058		243.0209	118.244
8	114.8929	47.7006		251.2342	133.0842
9	57.7454	45.0089		154.8948	117.3861
10	116.8558	42.5479		158.6874	76.0077
11	57.9053	37.0947		31.1516	27.4726
avg	63.1797	37.6075		123.8365	79.2115

	Coh 3	Coh 3		Coh 3	Coh 3
	Q Cond 12	Q Cond 3		M Cond 12	M Cond 3
sub 1	88.6811	57.7085		200.3328	117.4457
2	90.1203	27.9961		67.4388	48.8349
3	43.3968	37.7191		66.6153	76.2506
4	6.3232	7.6397		13.731	14.8339
5	61.2744	19.8993		62.2653	26.2395
6	30.7203	22.4266		112.0823	71.7124
7	144.9014	89.9731		240.146	135.6126
8	82.2978	79.6476		189.3693	124.5035
9	79.6051	67.8932		151.1834	133.2899

	10	72.1132	53.8075		98.142	86.2337
	11	71.079	46.159		43.14	27.6566
avg		70.0466	46.4427		113.1315	78.4194

		Coh 4	Coh 4		Coh 4	Coh 4
		Q Cond 12	Q Cond 3		M Cond 12	M Cond 3
sub 1		86.3976	27.7702		141.318	58.3029
	2	13.0306	0		13.0306	0
	3	76.2589	58.1052		78.308	76.5925
	4	35.997	5.9815		21.5852	6.138
	5	34.6966	2.3702		12.6641	6.8603
	6	39.5596	16.8384		155.8936	67.1454
	7	73.0899	16.928		75.6431	24.1873
	8	102.5189	52.4599		143.063	88.0845
	9	114.5841	63.1916		69.1006	69.4292
	10	112.3316	82.939		159.4489	66.6557
	11	41.8455	25.5989		55.0669	81.0518
avg		66.3918	32.0166		84.102	49.4952
